# *RGA1* Negatively Regulates Thermo-tolerance by Affecting Carbohydrate Metabolism and the Energy Supply in Rice

**DOI:** 10.1186/s12284-023-00649-w

**Published:** 2023-07-26

**Authors:** Baohua Feng, Yongqiang Xu, Weimeng Fu, Hubo Li, Gengmi Li, Juncai Li, Wenting Wang, Longxing Tao, Tingting Chen, Guanfu Fu

**Affiliations:** 1 National Key Laboratory of Rice Biology, China National Rice Research Institute, Hangzhou, 310006 China; 2 Key Laboratory of Southwest Rice Biology and Genetic Breeding, Ministry of Agriculture/Luzhou Branch of National Rice Improvement Center, Rice and Sorghum Research Institute, Sichuan Academy of Agricultural Sciences, Deyang, China; 3 China National Key Laboratory of Rice Biology, Jilin Agricultural University, Changchun, 130118 Jilin China

**Keywords:** *Oryza sativa* L., Heat stress, *RGA1*, Energy homeostasis, Carbohydrate metabolism

## Abstract

**Background:**

Signal transduction mediated by heterotrimeric G proteins, which comprise the α, β, and γ subunits, is one of the most important signaling pathways in rice plants. *RGA1*, which encodes the Gα subunit of the G protein, plays an important role in the response to various types of abiotic stress, including salt, drought, and cold stress. However, the role of *RGA1* in the response to heat stress remains unclear.

**Results:**

The heat-resistant mutant *ett1* (*enhanced thermo-tolerance 1*) with a new allele of the *RGA1* gene was derived from an ethane methyl sulfonate-induced Zhonghua11 mutant. After 45 °C heat stress treatment for 36 h and recovery for 7 d, the survival rate of the *ett1* mutants was significantly higher than that of wild-type (WT) plants. The malondialdehyde content was lower, and the maximum fluorescence quantum yield of photosystem II, peroxidase activity, and *hsp* expression were higher in *ett1* mutants than in WT plants after 12 h of exposure to 45 °C. The RNA-sequencing results revealed that the expression of genes involved in the metabolism of carbohydrate, nicotinamide adenine dinucleotide, and energy was up-regulated in *ett1* under heat stress. The carbohydrate content and the relative expression of genes involved in sucrose metabolism indicated that carbohydrate metabolism was accelerated in *ett1* under heat stress. Energy parameters, including the adenosine triphosphate (ATP) content and the energy charge, were significantly higher in the *ett1* mutants than in WT plants under heat stress. Importantly, exogenous glucose can alleviate the damages on rice seedling plants caused by heat stress.

**Conclusion:**

*RGA1* negatively regulates the thermo-tolerance in rice seedling plants through affecting carbohydrate and energy metabolism.

**Supplementary Information:**

The online version contains supplementary material available at 10.1186/s12284-023-00649-w.

## Background

Rice (*Oryza sativa* L.) is the major staple food for more than half of the world’s population. Rice plants are always exposed to various types of abiotic stress, such as heat, cold, and drought stress. Heat stress always occurs when the temperature exceeds the threshold beyond which plants can tolerate for an extended period (Sehgal et al. 2019; Li et al. 2020; Jajoo and Allakhverdiev 2017; Kumazaki and Suzuki 2019). Under such stress, the thermolabile proteins was inactivated, and that induces the accumulation of reactive oxygen species and programmed cell death (Grover et al. 2013; Liu et al. 2016; Zhang et al. 2016), which causes irreversible damage to crop plants. It has been reported that a large decrease in spikelet fertility, grain yield and quality were showed in those rice plants caused by heat stress at reproductive stage (Zhang et al. 2016, 2017 and 2018; Fu et al. 2015; 2016). Therefore, it is urgent to breed heat-resistant rice cultivar and develop heat-resistant cultivation technique to alleviate yield losses caused by extreme high temperature climates.

These years, several genes have been identified conferring heat tolerance in rice (Li et al. 2015a; Xu et al. 2020; Kan et al. 2022; Zhang et al. 2022). *Thermo-tolerance 1* (*OsTT1*), which encodes the α subunit of the 26S proteasome, plays a role in regulating the thermo-tolerance of African rice by eliminating cytotoxic denatured proteins (Li et al. 2015a). *SLENDER GUY 1* (*SLG1*) encodes the cytosolic tRNA 2-thiolation protein 2 and enhances thermo-tolerance in *indica* rice (Xu et al. 2020). It has been found that *OsTT2*, which encodes a Gγ subunit, confers rice thermo-tolerance via the SCT1 (sensing Ca^2+^ transcription factor 1)-dependent alteration of wax biosynthesis (Kan et al. 2022). Further, *OsTT3*, consisting of two genes, positive regulator *TT3.1* and the negative regulator *TT3.2*, that interact together to enhance rice thermotolerance (Zhang et al. 2022). However, how these genes affecting energy metabolism in rice plants under heat stress has not been documented in reference. As well-known, heat stress can disturb energy metabolism (Li et al. 2020; Jiang et al. 2020), and thus reduce plant productivity and stress tolerance (Dobrota 2006; Hashida et al. 2009; Chen et al. 2022; Li et al. 2023).

The energy levels of organisms need to be maintained sufficiently high to support their survival and growth. While photosynthesis is the main driver of plant productivity, cellular respiration (glycolysis, citrate cycle, and mitochondrial electron transport) is responsible for converting fixed carbon into energy used for growth and development. Nicotinamide adenine dinucleotide (NAD^+^) plays a central role in the electron transport chain (De and Van 2011), which is crucial for maintaining the flow of energy. NAD^+^ can be synthesized via two pathways: de novo synthesis, which involves aspartate and requires a minimum of five adenosine triphosphates (ATPs), and the salvage pathway, which only requires three ATPs at most (Hashida et al. 2009). Overconsumption of NAD^+^ destabilizes normal cell function, and its re-synthesis depletes the cellular ATP pool. A low energy level limits anabolism and disturbs normal cellular activities, resulting in cell damage and ultimately cell death. Furthermore, the ratio of NAD^+^/NADH plays a key role in regulating many redox reactions (Noctor et al. 2007). NAD^+^ and its metabolites can interact with signaling pathways, which is often overlooked but remains an important function of NAD^+^ and its metabolites (Hunt et al. 2004; Koch-Nolte et al. 2009). The NAD^+^ content of cells must be maintained at a constant level to maintain normal functioning (Wang et al., 2007). When plants are exposed to oxidative stress, the synthesis of poly (ADP-ribose) polymerases (PARPs) is induced. PARPs are synthesized from NAD^+^, using large negatively charged chains of ADP-ribose to modify nuclear proteins such as histones and transcription factors. As a result, large numbers of NAD^+^ molecules are metabolized, which ultimately results in cell death (De et al. 2005). Under stress conditions, energy consumption is sharply increased, and energy production is inhibited; leading to negative effects on energy homeostasis. When energy levels decrease below a certain threshold, growth ceases, damage accumulates, and the organism ultimately dies. Thus, maintaining energy homeostasis is a major challenge for all living organisms, and there is an intimate relationship between energy availability and stress tolerance in plants (De and Van 2011; Dröge-Laser and Weiste 2018; Jiang et al. 2020). Under stress conditions, energy homeostasis can be maintained for a prolonged period by reducing the activity of PARPs, which enhances stress tolerance in *Arabidopsis thaliana* and *Brassica napus* (Amor et al. 1998; De et al. 2005).

The heterotrimeric G proteins, comprising the α, β, and γ subunits, perceive extracellular stimuli through cell surface receptors; they then transmit signals to effectors to initiate downstream biological processes (Gilman 1987). Unactivated G protein binds to the Gα subunit of GDP and combines with the Gβγ subunit to yield a dimer; when G protein is activated, the Gα subunit dissociates from Gβγ and interacts with downstream effectors until Gα is hydrolyzed to GDP by GTPase (Assmann 2002; Jones and Assmann 2004; Perfus-Barbeoch et al. 2004; Urano et al. 2013; Ferrero-Serrano et al. 2018; Yu et al. 2018). These processes include responses to hormones, drought, pathogens, and other developmental events (Wettschureck and Offermanns 2005). A large heterotrimeric G protein family exists in the mammalian genome; for example, the human genome encodes 23 Gα, 5 Gβ, and 12 Gγ proteins (Jones and Assmann 2004; Bisht et al. 2015); in plants, the heterotrimeric G protein repertoire in plants is much less diverse (Trusov et al. 2012). The rice genome contains a common Gα gene, *RGA1* (Ishikawa et al. 1995); a Gβ gene, *RGB1* (Ishikawa et al. 1996); four Gγ subunit genes, *RGG1*, *RGG2*, *GS3*, and *DEP1* (Kato et al. 2004; Fan et al. 2006; Huang et al. 2009; Zhang et al. 2019); and a fifth related Gγ gene or pseudogene *OsGGC2* (Botella 2012). Kan et al. (2022) reported that *OsTT2*, which encodes a Gγ subunit, confers rice thermo-tolerance through an SCT1 (sensing Ca^2+^ transcription factor 1)-dependent alteration of wax biosynthesis. Rice *RGA1* plays an important role in the response to various types of abiotic stress including salt, drought, and cold stress (Ferrero-Serrano and Assmann 2016; Jangam et al. 2016). However, the role that *RGA1* plays in sensing heat stress remains unclear.

In our previous study, a dwarf mutant that is sensitive to low-light was screened and identified; gene mapping and sequencing revealed that it is a new *RGA1* allelic mutant (Li et al. 2023). However, we found that this mutant is more resistant to extreme heat stress, which has not been reported before. Here, we compared the agronomic characters of this mutant, named *ett1*(*enhanced thermo-tolerance 1*), with wild-type (WT, Zhonghua11) plants. RNA sequencing (RNA-seq) was conducted to clarify the molecular mechanism by which *ett1* responds to heat stress. The malondialdehyde (MDA) content, the maximum fluorescence quantum yield of photosystem II (Fv/Fm), antioxidant capacity, carbohydrate content, and energy status were characterized to clarify the thermo-tolerance and mechanism underlying the response of *ett1* mutants to heat stress.

## Materials and Methods

### Plant Materials and Growth Conditions

The dwarf and rounded grain mutant was obtained from mutagenesis of the WT cultivar Zhonghua11 using ethane methyl sulfonate (Li et al. 2023). This mutant was selfed for more than six generations, and the target trait has been stably expressed under greenhouse and field conditions in Hangzhou, Zhejiang, China. Gene mapping and sequencing revealed that *ett1* is a new *RGA1* allelic mutant (Additional file 5: Table S1 and Additional file 1: Fig. S1). We compared the agronomic characters of *ett1* and wild-type (WT, Zhonghua11) plants (Additional file 2: Fig. S2). The *ovD1* (over-expressed D1 in *ett1* mutant) plant was selected from the over-expression lines and the original name was OE-1 (Li et al. 2023). To evaluate the thermo-tolerance of the mutant, the seeds of the WT and *ett1* mutants were directly sown in pots (10 cm in height and 10 cm in diameter) in a plant growth chamber at 30/23 ºC (day/night) under natural sunlight and 70–80% relative humidity until the seedlings reached the fifth- to sixth-leaf stage. The plants were then divided into two groups. One group was subjected to heat stress conditions (45 ºC) for 36 h, followed by recovery for 7 d; the other group was exposed to control conditions. After 12 h of heat stress treatment, the second expanded leaves were sampled, immediately frozen in liquid nitrogen, and stored at − 80ºC for analyzing RNA sequencing, Fv/Fm, MDA, antioxidant capacity, carbohydrates content, and energy status.

According to the previous results, sugar signaling might play a key role in mediating the heat response between WT and *ett1* seedling plants. Therefore, 0.1% glucose was sprayed onto rice seedling plants about 30 min before heat stress conducted. After 12 h of heat stress treatment, the second expanded leaves were sampled to determine the Fv/Fm, MDA, and the energy status.

### Maximum Fluorescence Quantum Yield of Photosystem II (Fv/Fm)

Fv/Fm measurements were conducted following a previously described method (Zhao et al. 2017). After 12 h of heat stress treatment, the leaves were adapted to the dark for 30 min. A portable chlorophyll fluorometer, PAM-2500 (Walz Heinz GmbH, Effeltrich, Germany), was then used to measure Fv/Fm.

### RNA Sequencing and Analysis

Total RNA was extracted from 100 mg leaves using the Trizol reagent (Invitrogen, CA, USA) following the manufacturer's protocol. Paired-end sequencing was conducted on an Illumina Hiseq 4000 system (LC Sciences, USA) per the manufacturer’s protocol. Prior to assembly, low-quality reads were removed. A total of 77.57 G of cleaned paired-end reads were produced. FPKM was calculated to evaluate the expression levels of genes. Differentially expressed genes (DEG) were identified according to the following criteria using the R package Ballgown: log_2_(fold change) > 1 or log_2_(fold change) < − 1 and *p*-value < 0.05. Gene Ontology (GO) analysis, Kyoto Encyclopedia of Genes and Genomes (KEGG) analysis, and Gene Set Enrichment Analysis (GSEA) 4.1.0, https://www.gsea-msigdb.org/gsea/ index.jsp) were conducted to analyze the sequencing data.

### Quantitative RT-PCR (qRT-PCR) Analysis

qRT-PCR was conducted using the Step One Plus™ Real-Time PCR system (Thermo Fisher Scientific Biotechnology, Shanghai, China). The primers used are listed in Additional file 6: Table S2. PCR analysis and detection were performed as described previously (Feng et al. 2013). The 2^−∆∆CT^ method was used to analyze relative gene expression levels of the mean values of three replicates.

### Measurements of AMP, ADP, ATP, NAD(H), and NADP(H) Contents

Frozen leaves (0.2 g) were homogenized with 2 mL of perchloric acid in an ice bath and centrifuged for 10 min at 8,000 × *g*. Next, 2 mL of NaOH was added to the supernatant and centrifuged for 10 min at 8,000 × *g*. The supernatant was collected and placed in an ice bath for analysis. The ATP and ADP content was determined using the ATP and ADP assay kits, respectively, according to the manufacturer’s instructions (Comin Biotechnology Co., Ltd., Suzhou, China). The energy charge was calculated according to the formula: EC = (ATP + 0.5ADP)/(ATP + ADP + AMP).

NAD^+^, NADH, NADP^+^, and NADPH were extracted with 1 mL of 0.1 M HCl or 0.1 M NaOH following the method of Matsumura and Miyachi (1983). The NAD^+^ and NADH content was determined using an assay kit, and different kits were used to determine the NADP^+^ and NADPH content per the manufacturer’s instructions (Comin Biotechnology Co., Ltd., Suzhou, China).

### Measurement of Carbohydrate Content

The total soluble sugar content was determined using the sulfuric acid–anthrone colorimetric method with modifications (DuBois et al. 1956). Leaf samples (0.2 g) were homogenized with 10 ml of deionized water and then boiled for 30 min three times. The extract was filtered and treated with anthrone and 98% sulfuric acid; the mixture was then incubated in boiling water for 10 min. After cooling to room temperature, the absorbance was measured at 620 nm using a spectrophotometer. The starch content was determined using the sulfuric acid–anthrone colorimetric method (DuBois et al. 1956). The sediment of the extract filtered with the sugar was dried, weighed, and boiled with deionized water at 9.2 M and 4.6 M perchloric acid; the supernatant was used to determine the starch content. The total content of non-structural carbohydrates (NSCs) was calculated as the sum of the content of soluble sugars and starch. The method of glucose extraction was identical to that for total soluble sugar, and the content of glucose was determined following a previously described method with minor modifications (Zhang et al. 2018).

### Measurement of Antioxidant Enzyme Activities and MDA Content

Frozen leaves (0.2 g) were ground in liquid nitrogen and then homogenized in 5 mL of 50 mM sodium phosphate buffer (pH = 7.0). The homogenate was centrifuged at 10,000 × *g* for 15 min at 4 °C, and the supernatant was stored in aliquots at -20ºC for further analysis. Superoxide dismutase (SOD) activity was measured according to the degree of inhibition of the photoreduction of nitroblue tetrazolium as described by Giannopolitis and Ries (1977). Peroxidase (POD) activity was measured according to the conversion of guaiacol to tetraguaiacol, which was monitored at 470 nm, as previously described by Maehly and Chance (1955). Catalase (CAT) activity was measured following the previously described method of Aebi (1983) with some modifications. Ascorbate peroxidase (APX) activity was measured following the method of Bonnecarrère et al. (2011). The content of MDA was estimated by measuring the concentration of thiobarbituric acid-reactive substances (Dhindsa et al. 1981). Frozen leaves (0.2 g) were homogenized in 2 mL of 5% trichloroacetic acid. The absorbance was measured at 450, 532, and 600 nm. MDA was calculated using following the formula: C (μM) = 6.45(A532-A600) − 0.56A450.

## Results

### Alterations in Carbohydrate Metabolism and the Energy Supply Confer *ett1* with Thermo-tolerance

**Fig. 1:**
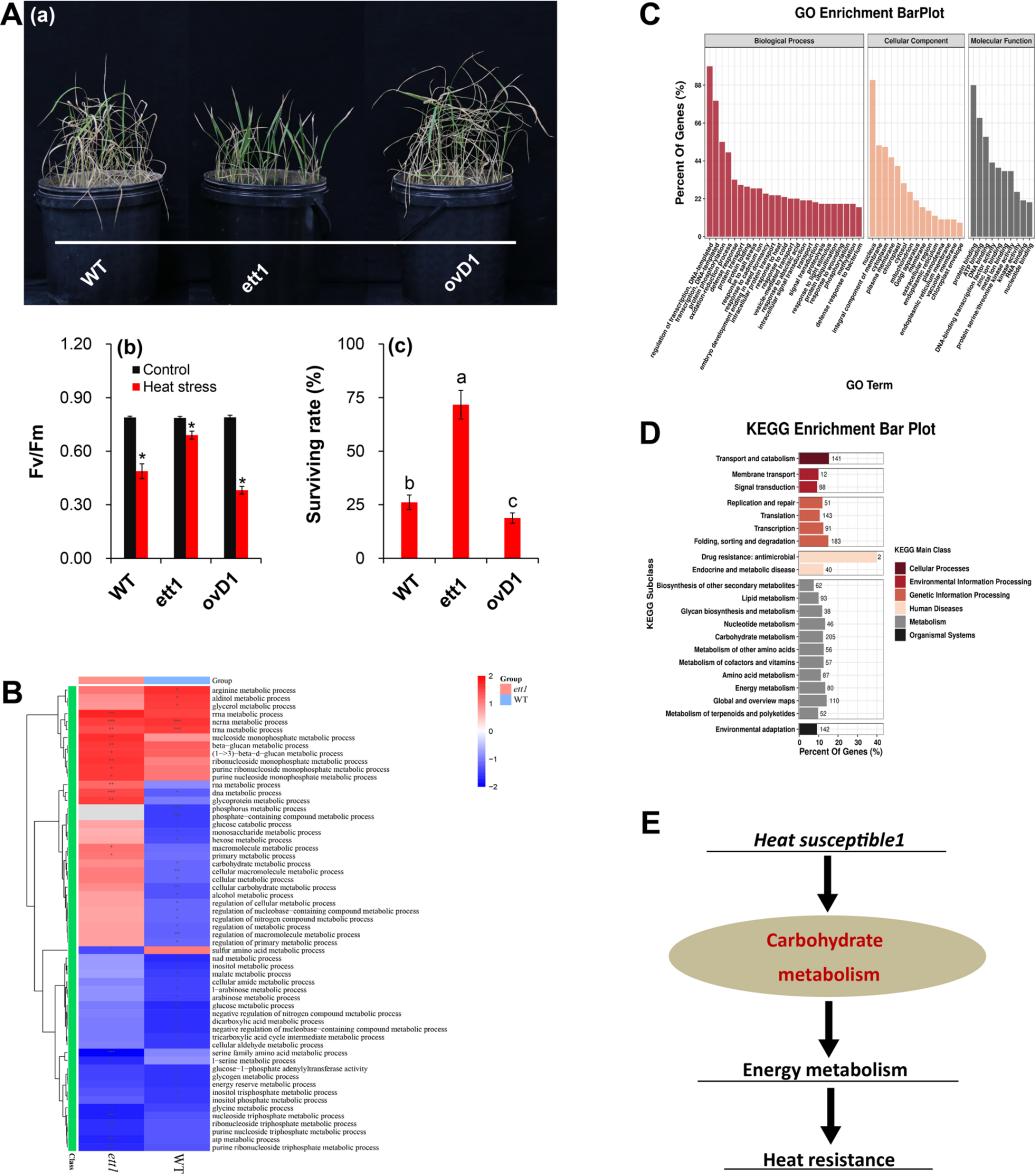
The performance of WT plants and ett1 mutants under HS and the results of RNA-seq analysis. **A** a, Plant performance under HS; Fv/Fm value (b) and survival rate (c) under HS; **B** GSEA of the RNA-seq data of WT plants and the ett1 mutant. **C** GO enrichment. **D** KEGG enrichment. **E** Simple descriptive model of the response of *ett1* mutants to HS. Fv/Fm, maximum fluorescence quantum yield of photosystem II; GSEA, Gene Set Enrichment Analysis; GO, Gene Ontology; KEGG, Kyoto Encyclopedia of Genes and Genomes; The arrow mark “→” indicates induction. Vertical bars denote standard deviations (*n* = 3). T-tests were conducted to evaluate the significance of differences among plants exposed to HS and control conditions. Different letters indicate significant differences under HS and control conditions (*P* < 0.05)

After rice seedling plants were exposed to 45 ºC for 36 h and allowed to recover for 7 d, most of the WT and *ovD1* leaves were withered, while most of leaves in the *ett1* mutants were still green (Fig. 1A a). Heat stress significantly decreased the values of Fv/Fm, in which the highest reduction was showed in *ovD1* plants, the next was WT plants, while the lowest reduction were presented in *ett1* mutants (Fig. 1A b). Accordingly, the highest survival rate was found in *ett1* mutants, followed by WT, and *ovD1* plants. This suggested that the gene *ett1* negatively regulated thermal tolerance in rice seedling plants (Fig. 1A c).

To clarify the mechanism underlying the heat response of *ett1*, RNA-seq was conducted under control conditions and heat stress treatment with WT and *ett1* seedling plants. The results of quality control analyses, DEG analyses, volcano plots, and heat map hierarchical clustering analysis are shown in the (Additional file 7: Table S3, Additional file 3: Fig. S3 and Additional file 4: S4). GSEA showed that the expression of genes involved in carbohydrate metabolic process, glucose metabolic process, glycogen metabolic process, NAD metabolic process, and energy reserve metabolism was down-regulated in WT plants under heat stress. The expression of genes involved in these five processes was less down-regulated in *ett1* mutants than in WT plants; in some cases, the expression of genes was up-regulated in *ett1* mutants (Fig. 1B). GO analysis was conducted on the four groups of plants. The GO terms response to heat and ATP binding were significantly enriched (Fig. 1C). Significantly enriched pathways according to KEGG analysis were protein processing in endoplasmic reticulum (ko04141), endocytosis (ko04144), RNA transport (ko03013), carbon metabolism (ko01200), and amino sugar and nucleotide sugar metabolism (ko00520) (Fig. 1 D). According to these findings, we speculate that carbohydrate metabolism and energy metabolism might be responsible for the significant differences in the response of the two rice seedling plants to heat stress (Fig. 1E).

**Fig. 2:**
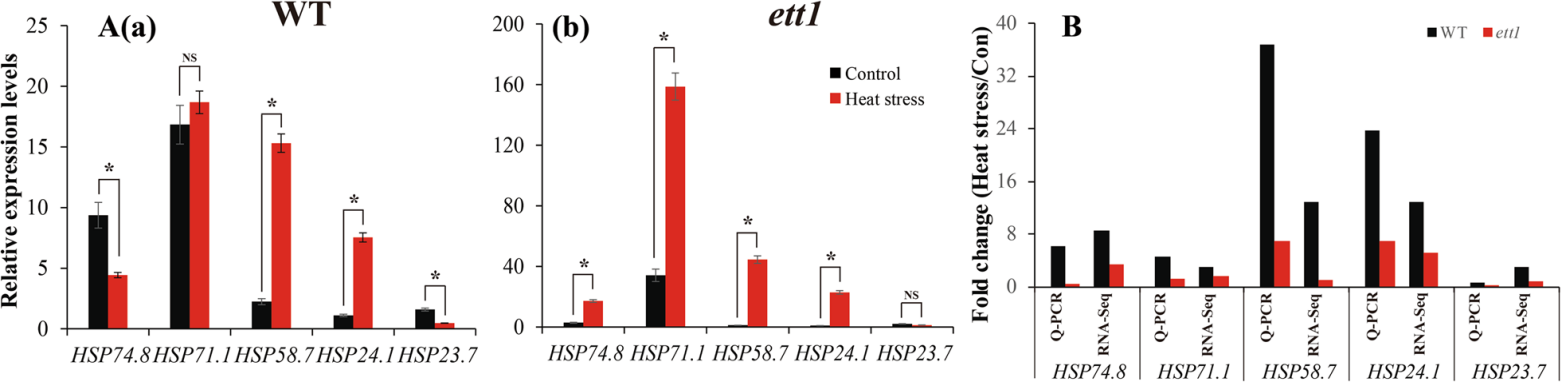
qRT-PCR validation of the differentially expressed transcripts identified by RNA-seq. Five *hsp* genes were selected for qRT-PCR analysis for validation. **A** qRT-PCR analysis of five hsp genes in WT (a) and *ett1* (b) plants. **B** Expression heat map of five *hsp* genes between WT and *ett1* mutants. *hsp*, heat shock protein; qRT-PCR, quantitative reverse transcription-polymerase chain reaction; *Hsp74.8*, LOC_Os09g29840; *Hsp71.1*, LOC_Os03g16860; *Hsp58.7*, LOC_Os09g31486; *Hsp24.1*, LOC_Os02g52150; *Hsp23.7*, LOC_Os12g38180. Vertical bars denote standard deviations (*n* = 3). T-tests were conducted to evaluate the significance of differences under HS and control conditions within a single genotype. “*” indicates significance difference exist, NS indicate no significance difference exist

Five heat shock protein (*Hsp*) genes were used to validate the RNA-seq data. The expression levels of genes were higher in *ett1* mutants than in WT plants, which indicates that the thermo-tolerance of the *ett1* mutants was greater than that of WT plants (Fig. 2A a and b). The RNA-seq results were similar to the qRT-PCR results (Fig. 2B).

### Carbohydrate Degradation is Affected by Heat Stress

**Fig. 3:**
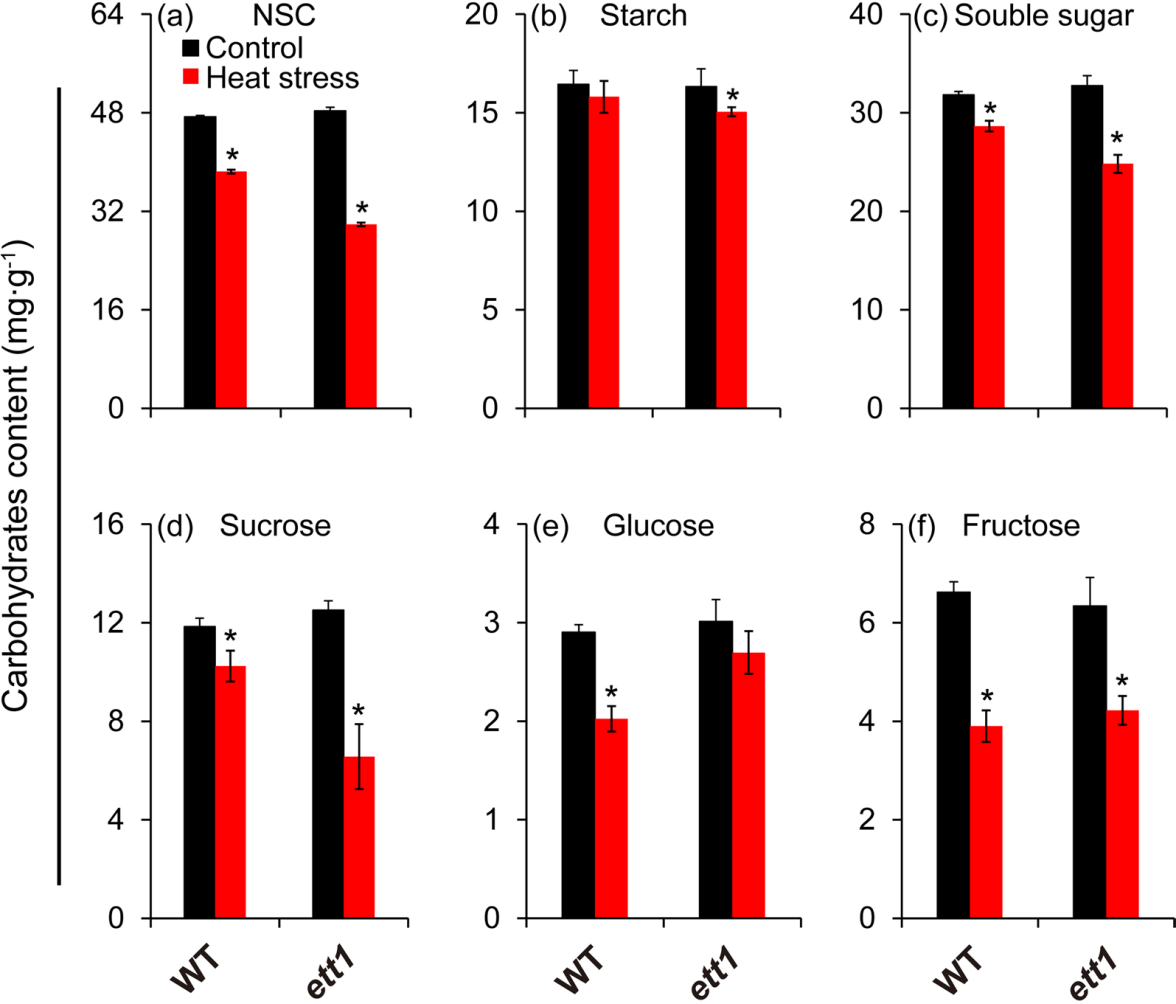
Effects of HS on carbohydrate metabolism in WT plants and *ett1* mutants. (a) NSC content. (b) Starch content. (c) Soluble sugar content. (d) Sucrose content. (e) Glucose content. (f) Fructose content. NSC, nonstructural carbohydrate. Vertical bars denote standard deviations (*n* = 3). T-tests were conducted to evaluate differences in the content of carbohydrates under HS and control conditions within a single genotype. “*” indicate significant differences between HS and control conditions (*P* < 0.05)

Sugar metabolism was significantly altered in *ett1* mutants under heat stress. The NSC, soluble sugar, starch, and sucrose content was significantly lower under heat stress than under control conditions in both *ett1* and WT plants, with the exception of the soluble sugar content in WT (Fig. 3). The NSC, starch, and sucrose content was 38.16%, 24.23%, and 47.52% lower in *ett1* mutants under heat stress than under control conditions, respectively; in WT plants, the NSC, starch, and sucrose content was 18.78%, 9.96%, and 13.54% lower under heat stress than under control conditions, respectively (Fig. 3 a, b, and d). Also, the decreases in the soluble sugar content under heat stress were much more pronounced in *ett1* mutants than in WT plants (Fig. 3c). Significant decrease in the glucose content was observed in WT plants under heat stress, while no significant difference was observed in glucose content in *ett1* mutants between heat stress and control conditions (Fig. 3e). Similar decreases were observed in fructose content under heat stress in *ett1* and WT plant (Fig. 3f).

**Fig. 4:**
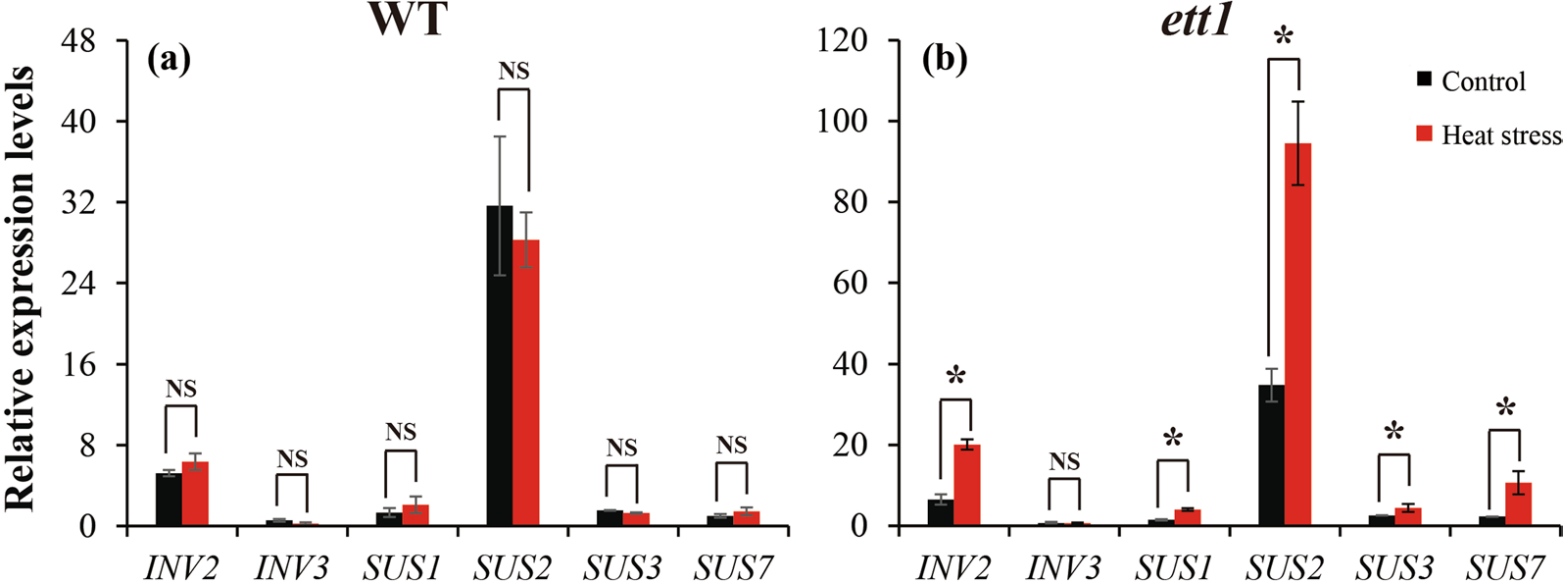
Effects of HS on the expression of sucrose synthase and invertase genes in WT plants (a) and *ett1* mutants (b). qRT-PCR, quantitative reverse transcription-polymerase chain reaction; SUS, sucrose synthase; INV, invertase; *INV2*, LOC_Os04g45290; *INV3*, LOC_Os02g01590; *SUS1*, LOC_Os03g28330; *SUS2*, LOC_Os06g09450; *SUS3*, LOC_Os07g42490; *SUS7*, LOC_Os04g17650. Vertical bars denote standard deviations (*n* = 3). T-tests were conducted to evaluate differences in the content of carbohydrates under HS and control conditions within a single genotype. “*” indicate significant differences between HS and control conditions (*P* < 0.05)

The expression patterns of six genes involved in sucrose metabolism were analyzed. The expression of the key regulatory genes involved in sucrose metabolism, including *invertase* (*INV)* and *sucrose synthase* (*SUS*), was up-regulated in *ett1* mutants under heat stress compared with control conditions (Fig. 4b); however, different patterns of gene expression were observed in WT plants. Specifically, the expression of *INV2*, *SUS1*, and *SUS7* was up-regulated under heat stress, and the expression of *INV3*, *SUS2*, and *SUS3* was down-regulated under heat stress (Fig. 4a). Increases in the expression of genes in *ett1* mutants were more pronounced than those observed in WT plants (Fig. 4).

### More ATP was Produced in *ett1* Mutants Under Heat Stress

**Fig. 5:**
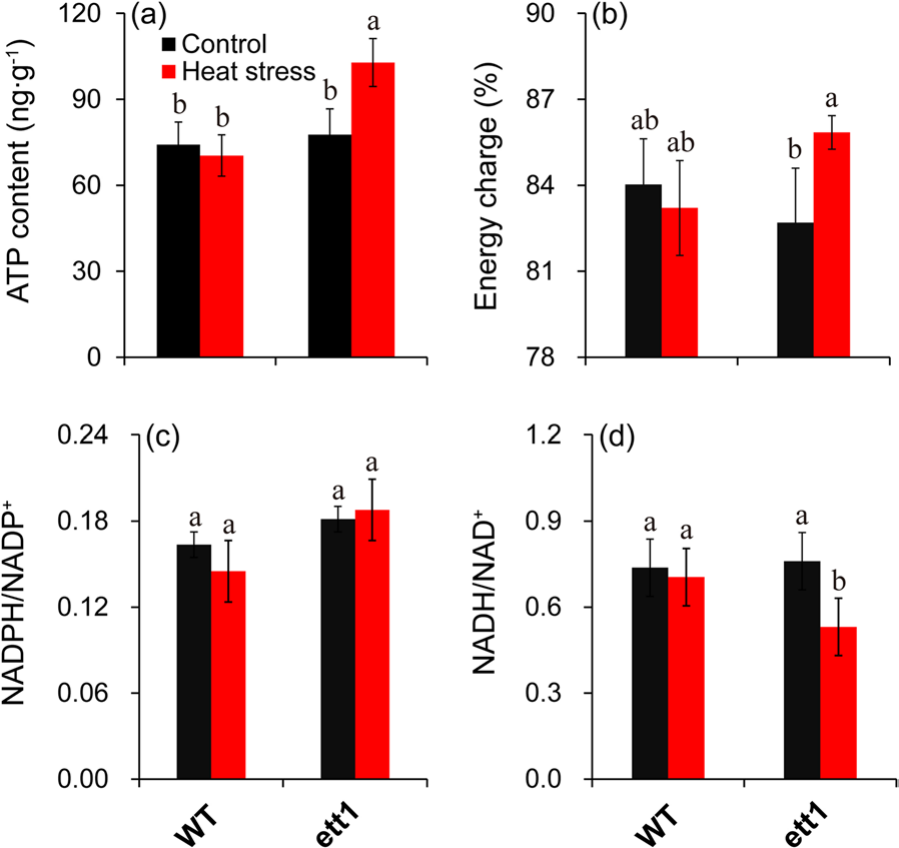
Effects of HS on energy metabolism in WT plants and *ett1* mutants. (a) ATP content. (b) Energy charge. (c) NADPH/NADP+. (d) NADH/NAD+. ATP, adenosine triphosphate; NADP (H), nicotinamide adenine dinucleotide phosphate; NAD(H), nicotinamide adenine dinucleotide. Vertical bars denote standard deviations (*n* = 3). T-tests were conducted to evaluate the significance of differences in energy metabolism variables under HS and control conditions within single genotypes. Different letters indicate significant differences between HS and control conditions (*P* < 0.05)

The ATP content and energy charge were significantly higher in *ett1* mutants under heat stress than under control conditions; while no significant difference was observed in the ATP content and energy charge in WT plants under heat stress and control conditions (Fig. 5a and b). No significant difference was observed in the ratio of NADPH/NADP^+^ in *ett1* mutants and WT plants between heat stress and control conditions (Fig. 5c). The ratio of NADH/NAD^+^ in *ett1* mutants was significantly decreased by heat stress, while no obvious difference between the heat stress and control were showed in WT plants (Fig. 5d).

### Antioxidant Capacity was not Decreased by Heat Stress in *ett1* Mutants

**Fig. 6:**
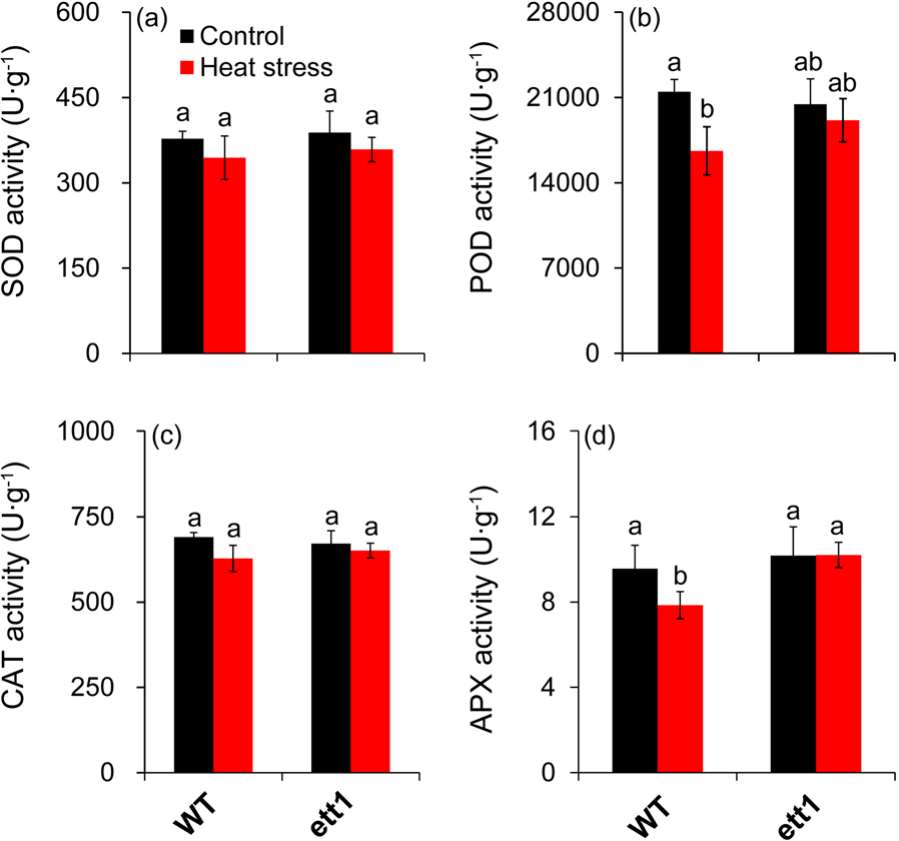
Effects of HS on the activity of antioxidant enzymes in WT plants and *ett1* mutants. (a) SOD activity. (b) POD activity. (c) CAT activity. (d) APX activity. SOD, superoxide dismutase; POD, peroxidase; CAT, catalase; APX, ascorbate peroxidase. Vertical bars denote standard deviations (*n* = 3). T-tests were conducted to evaluate the significance of differences in antioxidant enzyme activities under HS and control conditions within single genotypes. Different letters indicate significant differences under HS and control conditions (*P* < 0.05)

The activities of four antioxidant enzymes were measured. No significant difference was observed in SOD and CAT activities between the control and heat stress in both rice seedling plants (Fig. 6a and c). Under heat stress, notable decreases in POD and APX activity were presented in WT plants; in *ett1* mutants, no significant differences in POD and APX activity were observed under control conditions and heat stress (Fig. 6 b and d).

### Exogenous Glucose Application can Alleviate the Damage Induced by Heat Stress

**Fig. 7:**
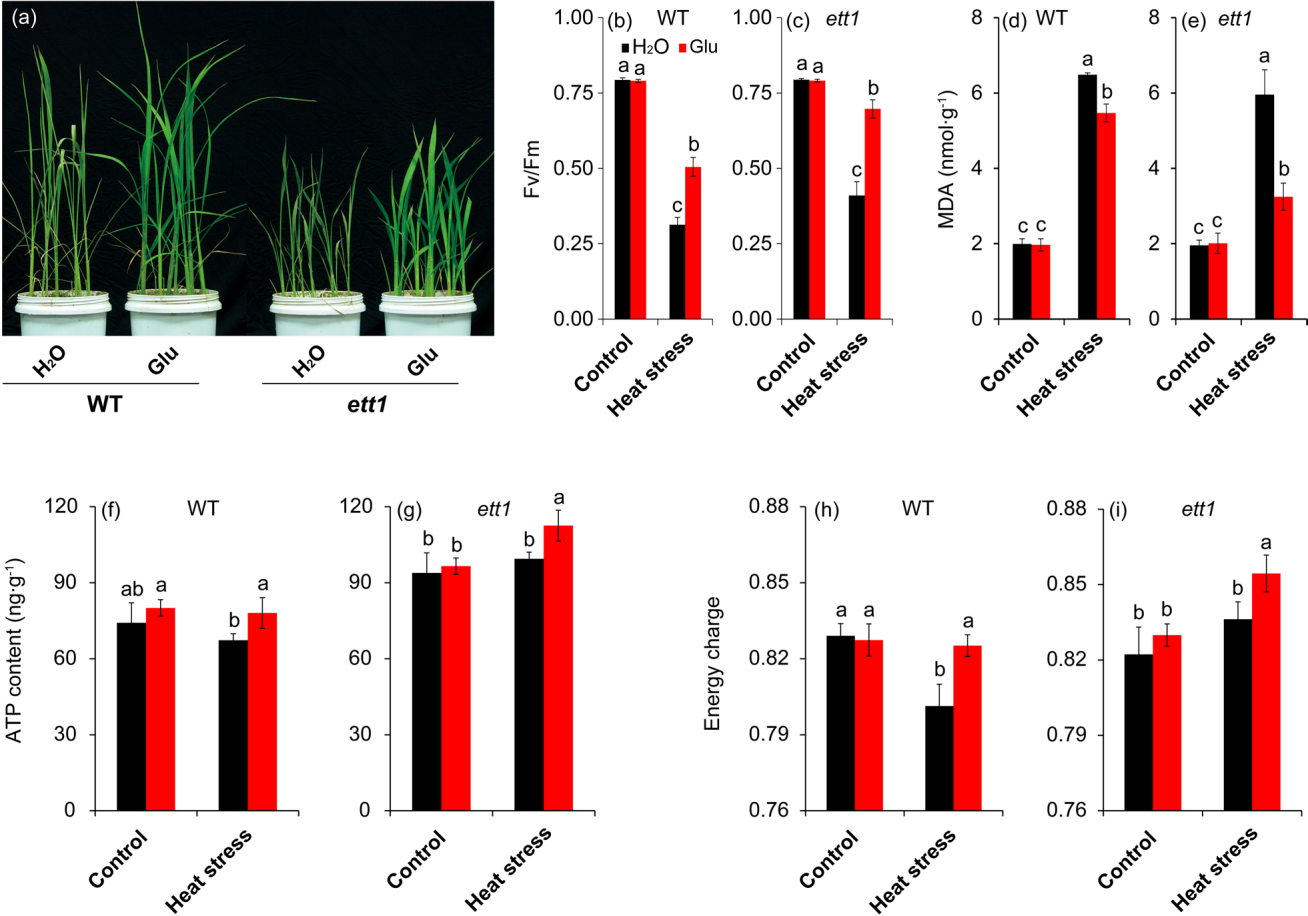
Exogenous glucose application alleviates damage caused by HS. (a) Photographs of leaves after spraying with glucose under HS. Fv/Fm value was alleviated by exogenous glucose in WT (b) and *ett1* (c). MDA content was alleviated by exogenous glucose in WT (d) and *ett1* (e). ATP content was affected by exogenous glucose in WT (f) and *ett1* (g). Energy charge was improved by exogenous glucose in WT (h) and *ett1* (i). MDA, Malondialdehyde; Fv/Fm, Maximum fluorescence quantum yield of PSII. Vertical bars denote standard deviations (*n* = 3). T-tests were conducted to evaluate the significance of differences under HS and control conditions within single genotypes. Different letters indicate significant differences under HS and control conditions (*P* < 0.05)

According to the results above, we inferred sugar signaling might play a key role in mediating the response of rice plants to heat stress, thus we conducted an exogenous glucose experiment to evaluate this possibility. Under control conditions, glucose caused little effects on the rice seedling plants compared with those plants treated with H_2_O; no obvious difference in Fv/Fm, MDA, ATP, and energy charge were found between both treatments in WT and *ett1* plants (Fig. 7). Leaves of both *ett1* mutants and WT plants sprayed with exogenous glucose were less curly than those sprayed with water (Fig. 7a). Heat stress significantly decreased the values of Fv/Fm of both rice plants, while this inhibition could be reversed by glucose (Fig. 7b and c). The MDA contents were obviously increased by heat stress, in particular for the WT; exogenous glucose significantly reduced the MDA content in either rice plants under such stress condition (Fig. 7d and e). In term of ATP and energy charge, notable enhancements were showed in the glucose treatment compared with the plants treated with H_2_O under heat stress (Fig. 7f–i).

## Discussion

The *ett1* mutant is a new allelic mutant of the D1 mutant, which has been largely documented in rice plants (Ishikawa et al. 1995; Fujisawa et al. 1999; Ashikari et al. 1999; Yang et al. 2014). Our previous results indicated that the *d1* mutants were susceptible to low light stress at anthesis, in which a large decrease in spikelet fertility were found in *d1* mutant plants compared with those of wide-type and over-expression lines caused by low-light stress (Li et al. 2023). Interestingly, this mutant displayed resistant to drought, salt and high light stress (Ferrero-Serrano and Assmann 2016; Ferrero-Serrano et al. 2018; Zait et al. 2021; Peng et al. 2019). Similarly, the *d1* plants display heat tolerance compared with its wide-type and over-expression lines (Fig. 1), suggesting that *ett1* negatively regulates thermal tolerance in rice plants. This finding was consistent with the results of Jangam et al. (2016), who considered that G-Protein Alpha Subunit played key roles in regulating multiple abiotic stresses such as drought, salinity, heat, and cold through microarray analysis. However, the mechanism underlying *ett1* affecting thermal-tolerance in rice plants has not been clarified.

GO and KEGG pathway analyses revealed that genes showing expression differences between the *ett1* mutants and WT plants were significantly enriched in carbohydrate and energy metabolism, which was inferred mainly contributing to their different heat tolerance (Fig. 1). As well-known, carbohydrates and energy metabolism not only molecule plant growth and development but also the response to various types of biotic and abiotic stress, including heat stress (Harker et al. 2009; Ruan 2014; Cabello et al. 2014; Li et al. 2015b; Islam et al. 2018). Indeed, *RGA1* has been reported to confer low-light resistance through improving the carbohydrate and energy metabolism in pistil of rice (Li et al. 2023). However, a larger decrease in NSC and sucrose were showed in *ett1* than WT plants under heat stress compared with their respective controls (Fig. 3). This was inconsistent with results that sucrose content of *RGA1* mutant was increased in pistil under low light conditions (Li et al. 2023), suggesting that inhibition on sucrose, rather than carbohydrates deficit contributed to the thermal tolerance showed in *ett1* plants (Jiang et al. 2020).

The invertase and sucrose synthesis are reported to play key roles in sucrose metabolism in plants (Sheen et al. 1999). Heat stress could reduce the activities of *CWINS* and *SUS*, deplete starch storage, reduce the hexose content in anther and pistil tissues, and ultimately result in pollen abortion (Ruan 2014; Li et al. 2015b). Similar results were found in this experiment, that increase in the relative expression levels of *SUS1*, *SUS2*, *SUS3*, *SUS7*, *INV2* and *INV3* were higher than those of WT plants under heat stress (Fig. 4). Therefore, it was inferred that *ett1* affected thermal-tolerance in rice plants through alleviating inhibition on expression of *INVs* and *SUSs* caused by heat stress. However, different roles in regulating heat response in plants were also found between the invertase and sucrose synthase (Jiang et al. 2020). Compared with heat-tolerant cultivar, lower expression of *sucrose phosphate synthase* (*SPS*) and *SUS* were found in heat-sensitive cultivar, whereas the expression levels *cell wall invertase* (*CWINs*) genes in heat–resistant cultivar were significantly lower than those in heat-susceptible line (Zhang et al. 2010). Further, *RGA1* can alleviate the inhibition on acid invertase activity in pistil of rice caused by low-light stress, while such effect was not presented in sucrose synthase activity (Li et al. 2023). Interestingly, low light stress obviously inhibited the genes of *SUS2*, *INV2*, and *CIN2* in pistil of *RGA1* mutant plants. Therefore, how *ett1* affecting sucrose metabolism in rice plants under heat stress required further researches.

Energy status is important for plants surviving in abiotic stress, since increases in antioxidant capacity and the accumulation of HSPs induced by high temperature and other adverse conditions require large amounts of energy; energy deficiencies and decreases in energy utilization efficiency can increase the stress sensitivity of plants (Zhang et al. 2017; Li et al. 2020). The present results indicated that energy status including ATP and energy charge were obviously increased by heat stress in *ett1* seedling plants compared with control, while no obvious difference in WT seedling plants were showed between the control and heat stress. This suggested that *ett1* affected the thermal tolerance in rice seedling plants by improving energy status, which could provide more energy for the strong antioxidant capacity and accumulation of heat shock proteins (Fig. 2 and Fig. 5). Indeed, the changes in contents of NADP(H) and NAD(H) also play key roles in regulating energy status in plants (Igamberdiev and Bykova 2023). There was obvious difference in NADPH/NADP^+^ between the control and heat stress in either rice plants; A notable decrease in NADH/NAD^+^ were presented in *ett1* mutant plants under heat stress compared with control (Fig. 5). This indicated that NADH/NAD^+^ was an important factor in mediating *ett1* to affected the energy homeostasis in plants under heat stress, which has been not reported previously.

These years, studies have indicated that, similar to classical plant hormones, sugar molecules not only a nutrient, but also can act as signaling molecules that control gene expression, developmental processes and response to abiotic stress in plants (Sheen et al. 1999; Wang et al. 2020; Choudhary et al. 2022). Heat stress significantly decreased the glucose content in wt seedling plants, while no obvious difference in *ett1* seedling plants were showed between the control and heat stress, suggesting that glucose signaling might be involved in the process of *ett1* regulating thermal tolerance. This hypothesis was confirmed by the exogenous glucose spraying onto rice plants under heat stress (Fig. 7), which might be related its function in enhancing the acid invertase activity as well as inactivating PARP to regulate the energy utilizing efficiency (Jiang et al. 2020).

## Conclusion

The *ett1* mutant has a dysfunctional *RGA1* gene that encodes the Gα subunit of the heterotrimeric G protein, and it negatively regulates thermal tolerance in rice. Under heat stress, the survival rate of *ett1* mutants was significantly higher than that of WT plants. According to the transcriptome analysis, the sugar and energy metabolism were involved in mediating *ett1* to affect the thermal tolerance. In this process, the ATP content and energy charge was obviously increased by heat stress in *ett1* plants, which mainly responsible for the antioxidant capacity and accumulation of HSPs. Exogenous glucose can alleviate heat damages on rice seedling plants through increasing the ATP content and energy charge. Thus, we concluded that *ett1* negatively regulated thermo-tolerance in rice by improving carbohydrate metabolism, glucose signaling and energy status.

## Supplementary Information

**Additional file 1.** The gene mapping, sequencing results and predicted protein coding of the mutant *ett1*.

**Additional file 2.** The performance of agronomic traits in the mutant *ett1* and WT.

**Additional file 3.** Number of DEGs in *ett1* and WT under heat stress and control condition.

**Additional file 4.** The hierarchical clustering analysis of the DEGs of the mutant *ett1* and WT under heat stress and control condition.

**Additional file 5.** Genetic analysis of the mutant *ett1*.

**Additional file 6.** Primer sequences used in the quantitative RT-PCR.

**Additional file 7.** The quality control analysis of the RNA-sequencing results.

## Data Availability

Information on plant materials are included in our previous article (Li et al. 2023). Other datasets supporting the conclusions of this article are included in the article (and its additional files).

## Abbreviations

APX: Ascorbate peroxidase
CAT: Catalase
*CWIN*: Cell wall invertase
Fv/Fm: Maximum fluorescence quantum efficiency
GO: Gene ontology
GSEA: Gene Set Enrichment Analysis
Hsp: Heat shock proteins
*INV*: Invertase
KEGG: Kyoto Encyclopedia of Genes and Genomes
MDA: Malondialdehyde
NAD: Nicotinamide adenine dinucleotide
NBT: Nitroblue tetrazolium
NSC: Non-structural carbohydrates
PCD: Programmed cell death
POD: Peroxidase
ROS: Reactive oxygen species
*SPS*: Sucrose phosphate synthase
SOD: Superoxide dismutase
*SUS*: Sucrose synthase
*SUT*: Sucrose transporters
