# Supplementary figures and images for: RGA1 Negatively Regulates Thermo-tolerance by Affecting Carbohydrate Metabolism and the Energy Supply in Rice
Source: Rice (N Y). 2023 Jul 26;16:32. doi: 10.1186/s12284-023-00649-w (PMC10371973; doi:10.1186/s12284-023-00649-w)

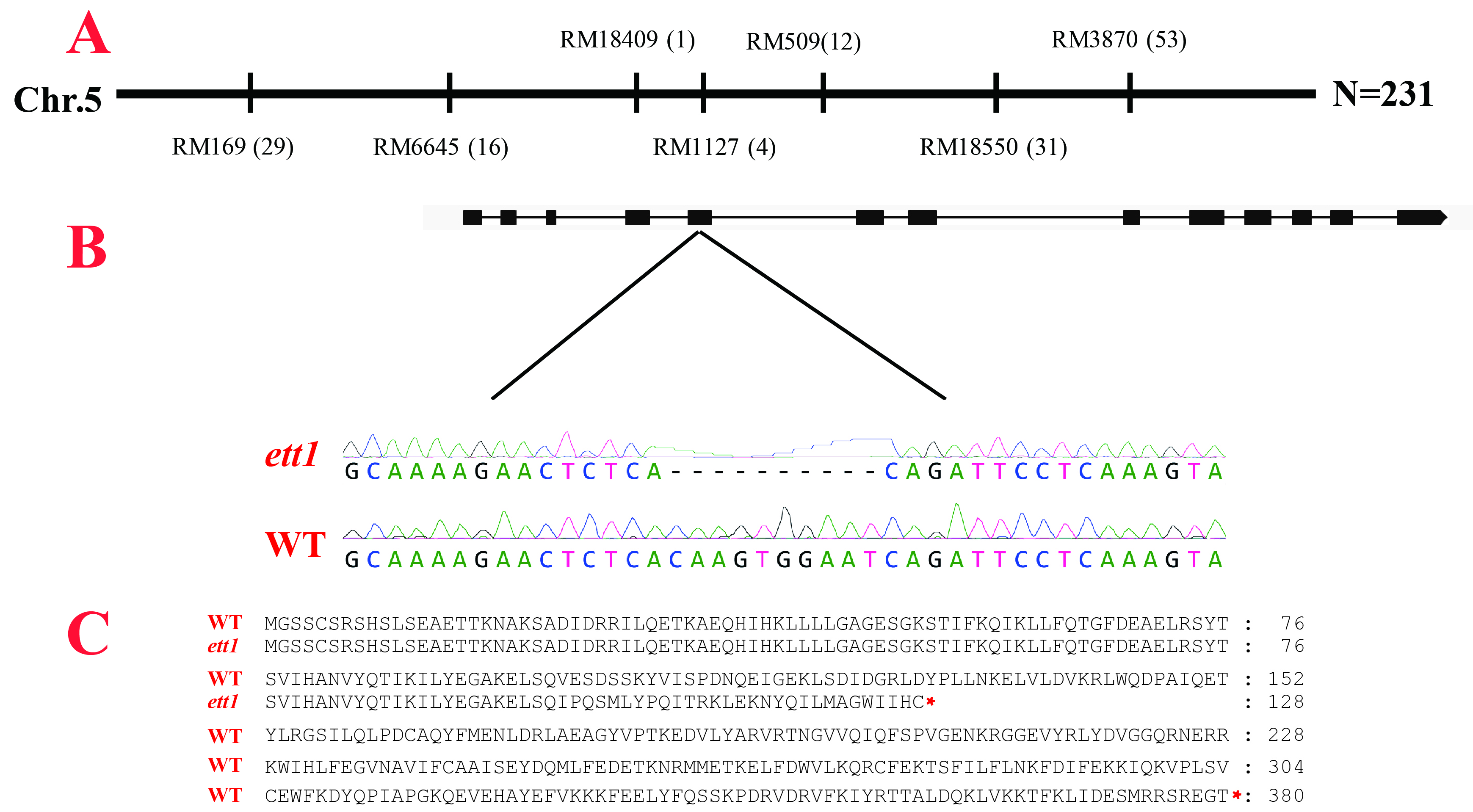

Supplement: Supplementary file 1 — Additional file 1. The gene mapping, sequencing results and predicted protein coding of the mutant ett1. [file 12284_2023_649_MOESM1_ESM.jpg]

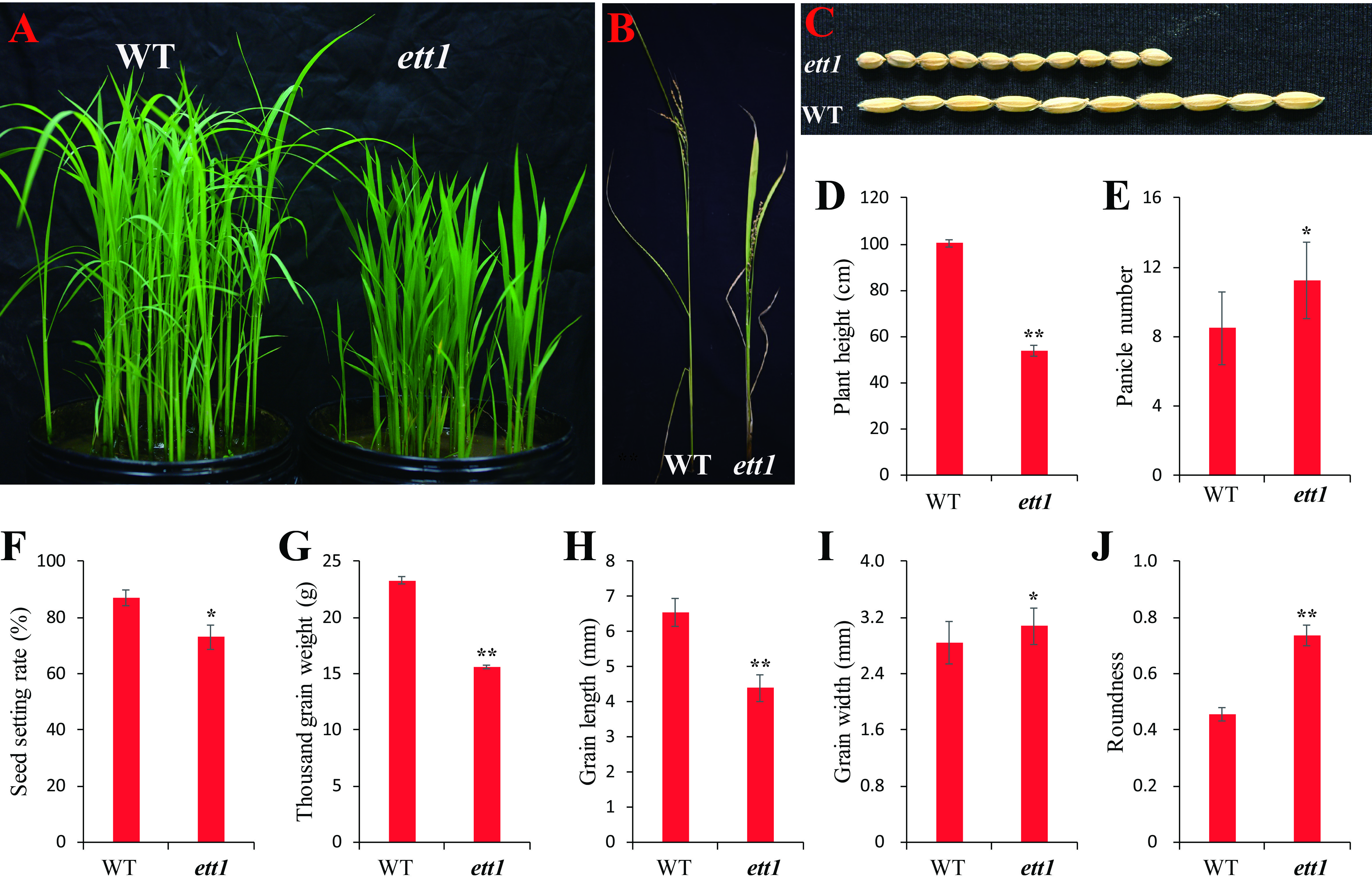

Supplement: Supplementary file 2 — Additional file 2. The performance of agronomic traits in the mutant ett1 and WT. [file 12284_2023_649_MOESM2_ESM.jpg]

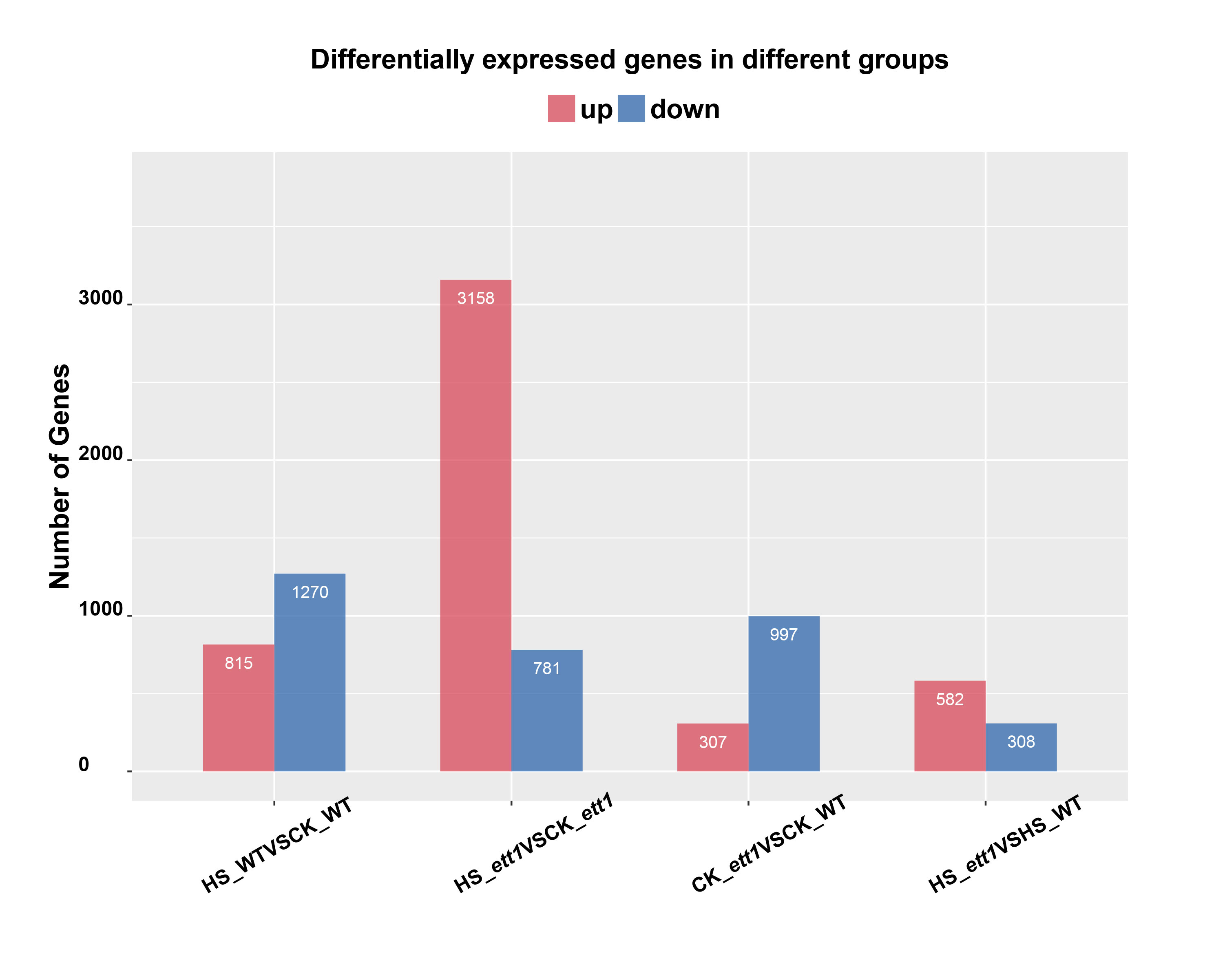

Supplement: Supplementary file 3 — Additional file 3. Number of DEGs in ett1 and WT under heat stress and control condition. [file 12284_2023_649_MOESM3_ESM.jpg]

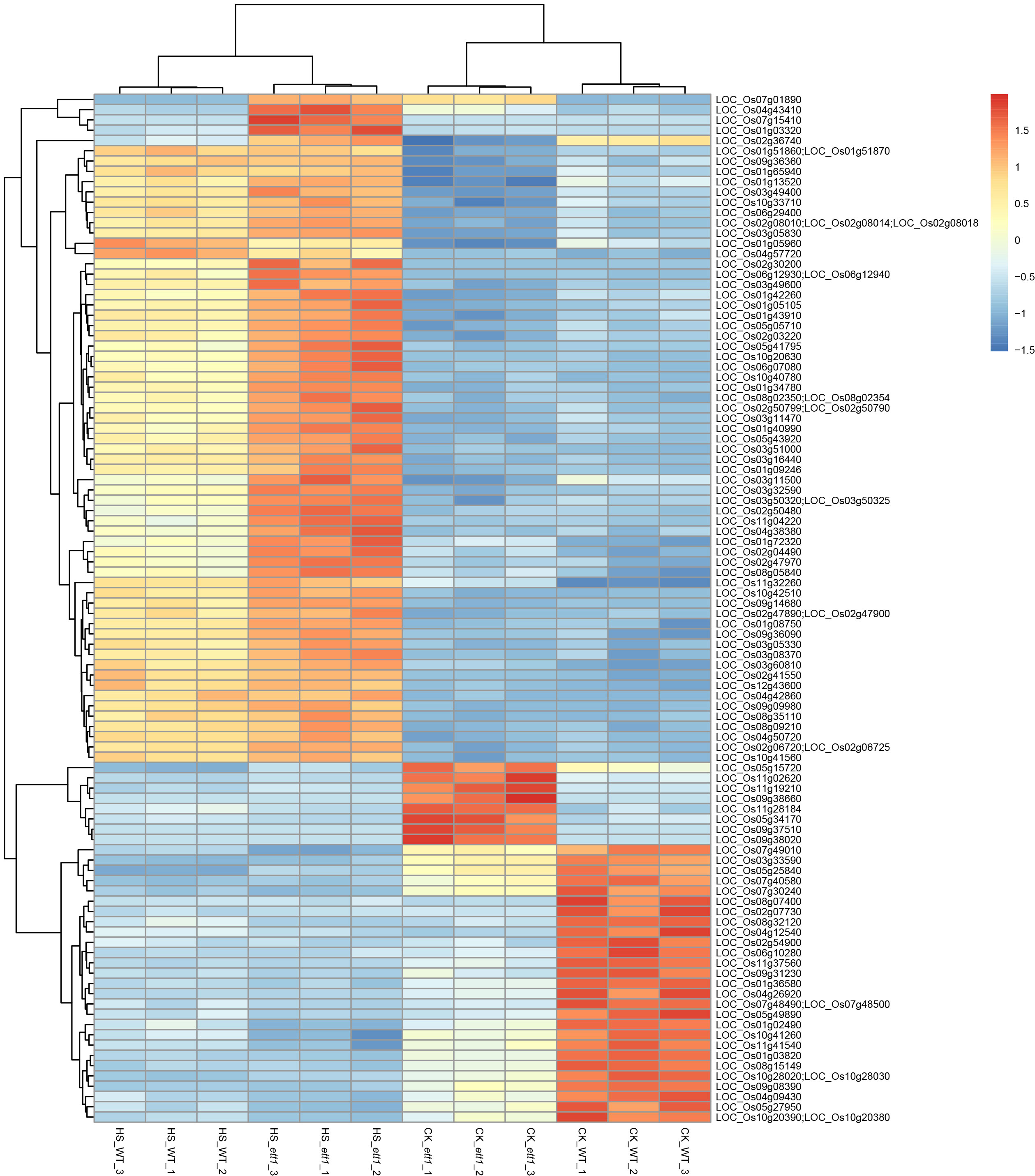

Supplement: Supplementary file 4 — Additional file 4. The hierarchical clustering analysis of the DEGs of the mutant ett1 and WT under heat stress and control condition. [file 12284_2023_649_MOESM4_ESM.jpg]

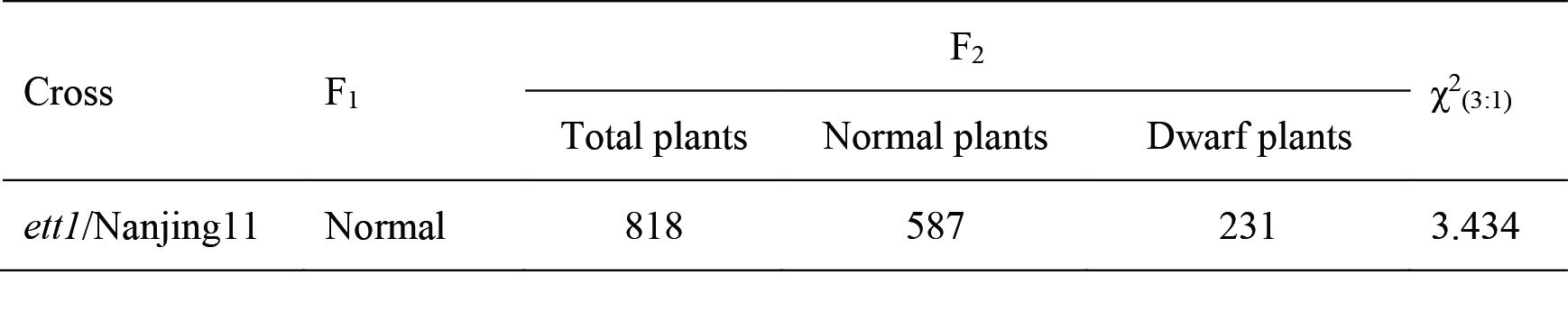

Supplement: Supplementary file 5 — Additional file 5. Genetic analysis of the mutant ett1. [file 12284_2023_649_MOESM5_ESM.jpg]

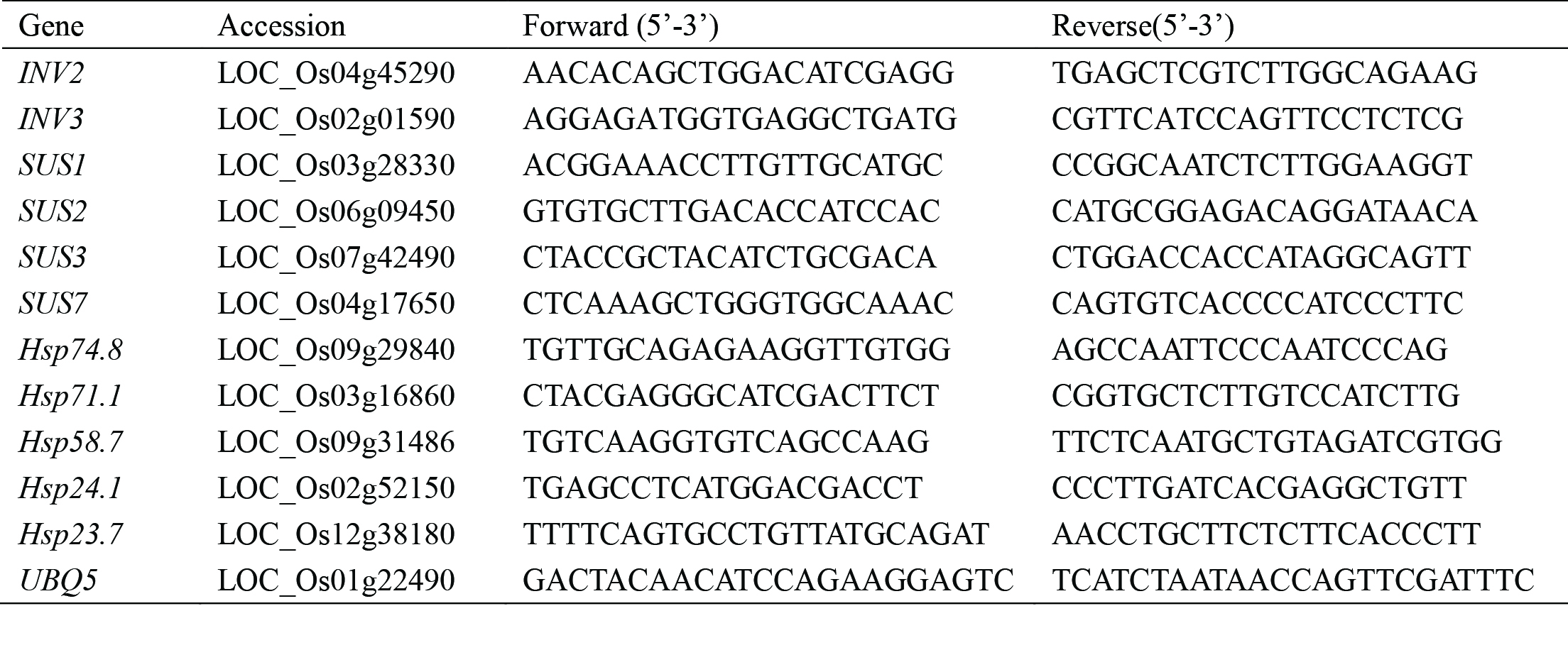

Supplement: Supplementary file 6 — Additional file 6. Primer sequences used in the quantitative RT-PCR. [file 12284_2023_649_MOESM6_ESM.jpg]

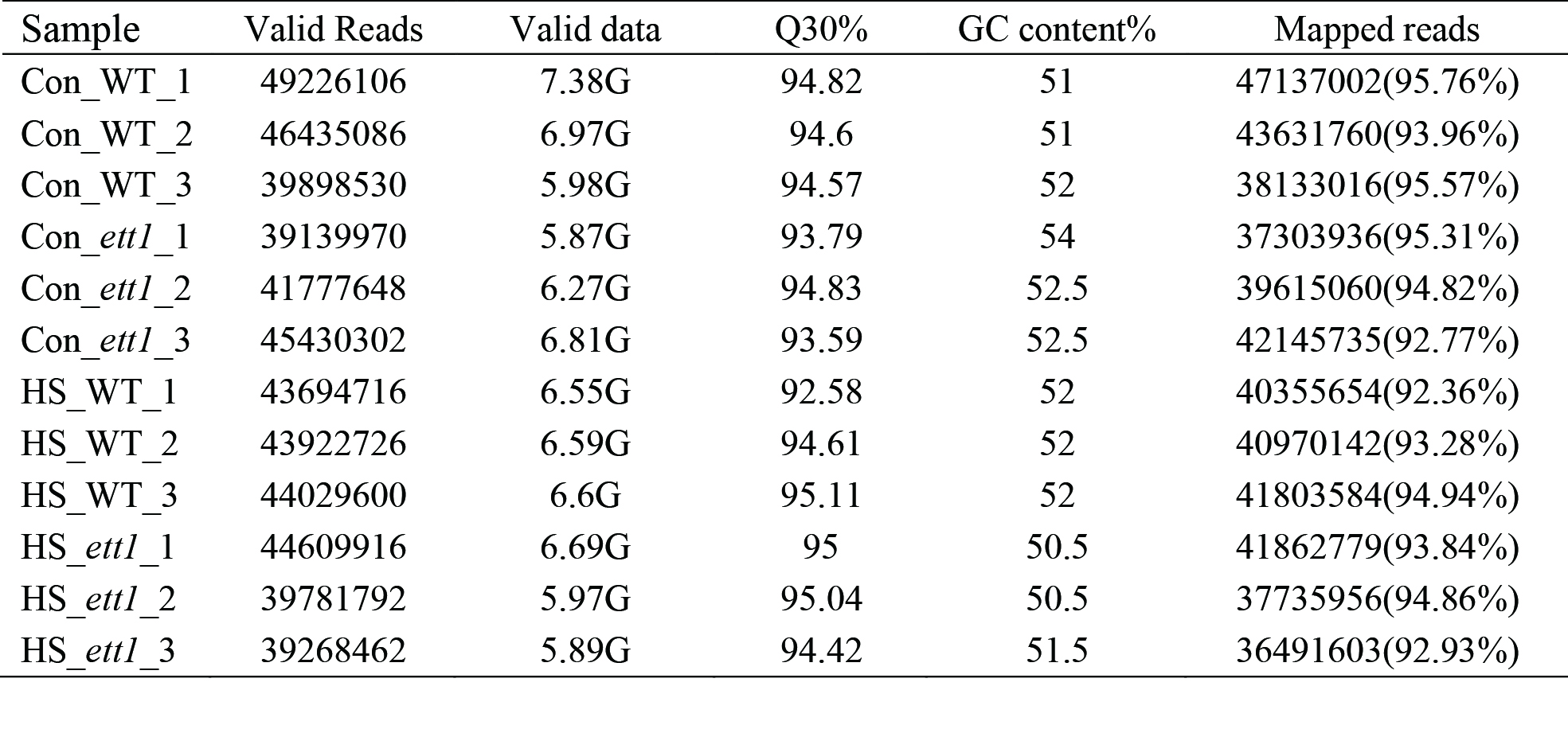

Supplement: Supplementary file 7 — Additional file 7. The quality control analysis of the RNA-sequencing results. [file 12284_2023_649_MOESM7_ESM.jpg]
